# Inhibition of return emerges with non-predictive spatial cueing of the stop-signal

**DOI:** 10.3389/fnhum.2025.1567597

**Published:** 2025-07-02

**Authors:** Md. Tanbeer Haque, Luca Conci, Giampiero Bardella, Sabrina Fagioli, Stefano Ferraina, Fabio Di Bello, Pierpaolo Pani

**Affiliations:** ^1^Department of Physiology and Pharmacology, Sapienza University, Rome, Italy; ^2^Behavioral Neuroscience PhD Program, Sapienza University, Rome, Italy; ^3^Department of Education, University of Roma Tre, Rome, Italy

**Keywords:** stop signal task, attention, inhibition of return, motor inhibition, cognitive control

## Abstract

The ability to suppress an inappropriate response can be influenced by several factors, including providing information on where to pay attention. For example, the spatial prediction of the stop signal location enhances inhibitory control in a Stop Signal Task. Here, we test whether a non-predictive spatial cueing improves inhibitory control as well. In this experiment, participants observed a vertical bar moving from a central position toward one of two circles on the screen. They were asked to press a key when the bar's motion was interrupted (go signal). In 25% of the trials (stop signal trials), after a variable delay following the go signal, a visual target (stop signal) appeared in one of the circles, requiring participants to inhibit their response to the go signal. In half of these trials, the stop signal appeared on the same side as the go signal (valid condition), and in the other half, it appeared on the opposite side (invalid condition). Our results show a facilitation effect for stop trials in the invalid condition compared to the valid condition, for targets occurring from 300 ms onward the go signal. This suggests an involvement of Inhibition of Return (IOR) in affecting the stop signal detection during motor control. Our findings provide new insights into the interaction between attentional processes and motor control, highlighting a temporally focused influence of exogenous attention in shaping motor inhibition.

## 1 Introduction

In everyday life, while driving, an animal might suddenly cross the road, forcing us to brake. If we are not paying attention, we may fail to detect the animal in time and stop quickly enough to avoid a potential danger to both the animal and ourselves. The ability to inhibit a response, also known as inhibitory control, is a fundamental mechanism for adaptive behavior, as it allows us to survive in a constantly changing environment (Wessel and Anderson, 2024). However, as illustrated in the previous example, inhibitory control can be influenced by attention. Indeed, the efficient visual detection of the animal is a necessary preliminary step to brake the car.

Various behavioral paradigms have been developed to study response inhibition, with the Stop Signal Task (Vince, 1948; Logan and Cowan, 1984; Mirabella et al., 2009; Montanari et al., 2017; Logan, 1981) being one of the most used. This task is well suited for investigating various factors influencing inhibitory control, such as the characteristics of the stop stimulus (van der Schoot et al., 2005; Montanari et al., 2017) and the complexity of the task (Middlebrooks et al., 2020). The possibility of receiving a reward for successful inhibition can also affect the effectiveness of inhibition (Boehler et al., 2012, 2014; Giamundo et al., 2021; Giuffrida et al., 2023).

The Stop Signal Task has been employed with various types of subjects, including children, adolescents (Albert et al., 2022), adults, and seniors (Paitel and Nielson, 2021). In some clinical populations, this task has been helpful in identifying slower inhibitory process, as evidenced by the Stop Signal Reaction Time (SSRT), which measures the latency of the inhibition process (Logan and Cowan, 1984; Logan, 1994; Menghini et al., 2018; Senkowski et al., 2023; Lipszyc and Schachar, 2010; Mar et al., 2022; Mirabella et al., 2020; Pani et al., 2013; see Section 2 for details). Additionally, this paradigm has been extensively used in neurophysiological research to examine how various brain regions contribute to inhibitory control (Hanes et al., 1998; Schall and Hanes, 1998; Paré and Hanes, 2003; Chen et al., 2010; Pani et al., 2014, 2018, 2022a; Giamundo et al., 2021; Brunamonti and Paré, 2023; Bardella et al., 2024a,b,c; Candelori et al., 2025).

In the Stop Signal Task, participants are required to respond as quickly as possible to a go signal in most of the trials (go trials). However, sometimes a stop signal is presented after the go signal (stop trials) following a delay, the Stop Signal Delay (SSD), instructing participants to inhibit their response (Verbruggen et al., 2019). The probability of response is influenced by the duration of this delay: shorter SSDs lead to a lower probability of response, while this probability increases progressively as the SSD becomes longer (Logan and Cowan, 1984). A task of this complexity involves a variety of cognitive processes, including decision making, attention and motor control (Verbruggen et al., 2014b). Nevertheless, some research considers motor control as a single, unified process, neglecting the impact of other cognitive functions during its functioning. Challenging this view, studies have demonstrated that attention can significantly influence motor inhibition. For example, the presence of distractors has been shown to impair the ability to inhibit responses (Verbruggen et al., 2014a). Moreover, inhibition is affected by spatial attention deployment, with improved performance observed when the stop signal is presented in a location already attended for a go signal (Hilt and Cardellicchio, 2020). Additionally, removing a fixation point, as it facilitates the disengagement of attention, impacts both the initiation and inhibition of movements. This process plays a critical role in modulating attentional shifts and movement inhibition (Fischer and Weber, 1993; Song and Nakayama, 2007; Mirabella et al., 2009). The relation between attention and inhibition is further corroborated by the fact that both processes may rely on overlapping neural networks, involving both cortical and sub-cortical regions (Corbetta et al., 2009; Aron et al., 2014; Alves et al., 2022). However, despite the existing studies in literature, many aspects of the relationship between attention and inhibition remain unexplored. For instance, the influence of spatial attention on motor control is not yet fully understood. Spatial attention can be involuntarily captured by salient, unexpected events (exogenous attention) or consciously directed to specific locations (endogenous attention).

In our previous work (Haque et al., 2024), we examined this relationship by administering a Stop Signal Task to human subjects, where a moving (from the center to the periphery) spatial cue indicated the probable location of the stop signal. More specifically, in stop trials, the cue predicted the location of the stop signal in valid trials (70%), while in invalid trials (30%), the stop signal appeared in the opposite position. Performance was significantly better in valid trials for stop signals appearing shortly after the go signal (up to 250 ms), suggesting that the cue influenced rapid exogenous attentional shifts (Mulckhuyse and Theeuwes, 2010). However, given the spatial predictability of the stop signal based on the task design, we cannot definitively rule out the possibility of an involvement of endogenous attention (Chica et al., 2014; Di Bello et al., 2019). To evaluate this possibility, we propose a task in which a non-predictive spatial cue was presented before the stop signal, ensuring an equal proportion of valid and invalid stops trials.

This task configuration is ideal for testing the effects of exogenous attention in motor control, including Inhibition of Return (IOR), a phenomenon in which accuracy decreases in valid trials compared to invalid trials when targets are presented with a delay of 300 ms or more after the peripheral cue (Posner et al., 1985; Klein, 2000). We found no facilitation effect on valid trials, indicating that the lack of spatial prediction heavily limited the impact of the stop signal in motor inhibition. However, accuracy was higher in invalid trials compared to valid trials at 300 and 400 ms from the presentation of the go signal (IOR), suggesting a participation of exogenous attention on motor control. Overall, our results complement previous findings, highlighting distinct effects of exogenous, and endogenous attention on action stopping.

## 2 Methods

### 2.1 Participants

Sixteen right-handed participants (eight males and eight females, mean age: 28.13 ± 6.55 years) with normal or corrected-to-normal vision participated in this study. We tested the independence assumption as a recruitment criterion. This assumption, defined by the Independent Race Model (Logan and Cowan, 1984), is crucial for obtaining a reliable estimate of Stop Signal Reaction Time (SSRT). The Independent Race Model describes stop trial performance as a competition between two independent processes: the GO process, triggered by the go signal, and the STOP process, initiated by the stop signal. The participant's behavior during stop trials depends on which process concludes first. A faster GO process leads to a response, while a quicker STOP process successfully inhibits the response (Logan and Cowan, 1984). The two processes are postulated to be independent, that is, their timing varies stochastically but without influencing each other. When this assumption is verified, the mean reaction times (RT) in wrong stop trials are expected to be shorter than those in go trials, on average (Verbruggen et al., 2019). All participants met this criterion (see Section 2.4). Ethical approval for the study was obtained from the Ethics Committee of “Roma Tre” University, and all procedures adhered to the Declaration of Helsinki. Each participant provided informed consent before taking part in the study.

### 2.2 Experimental design

In the classic version of the Stop Signal Task, two types of trials are presented: go trials, where the subject must respond as quickly as possible, and stop trials, where, after the presentation of a go signal, a stop signal appears following a certain delay (Stop Signal Delay), requiring the subject to inhibit their response.

**Figure 1:**
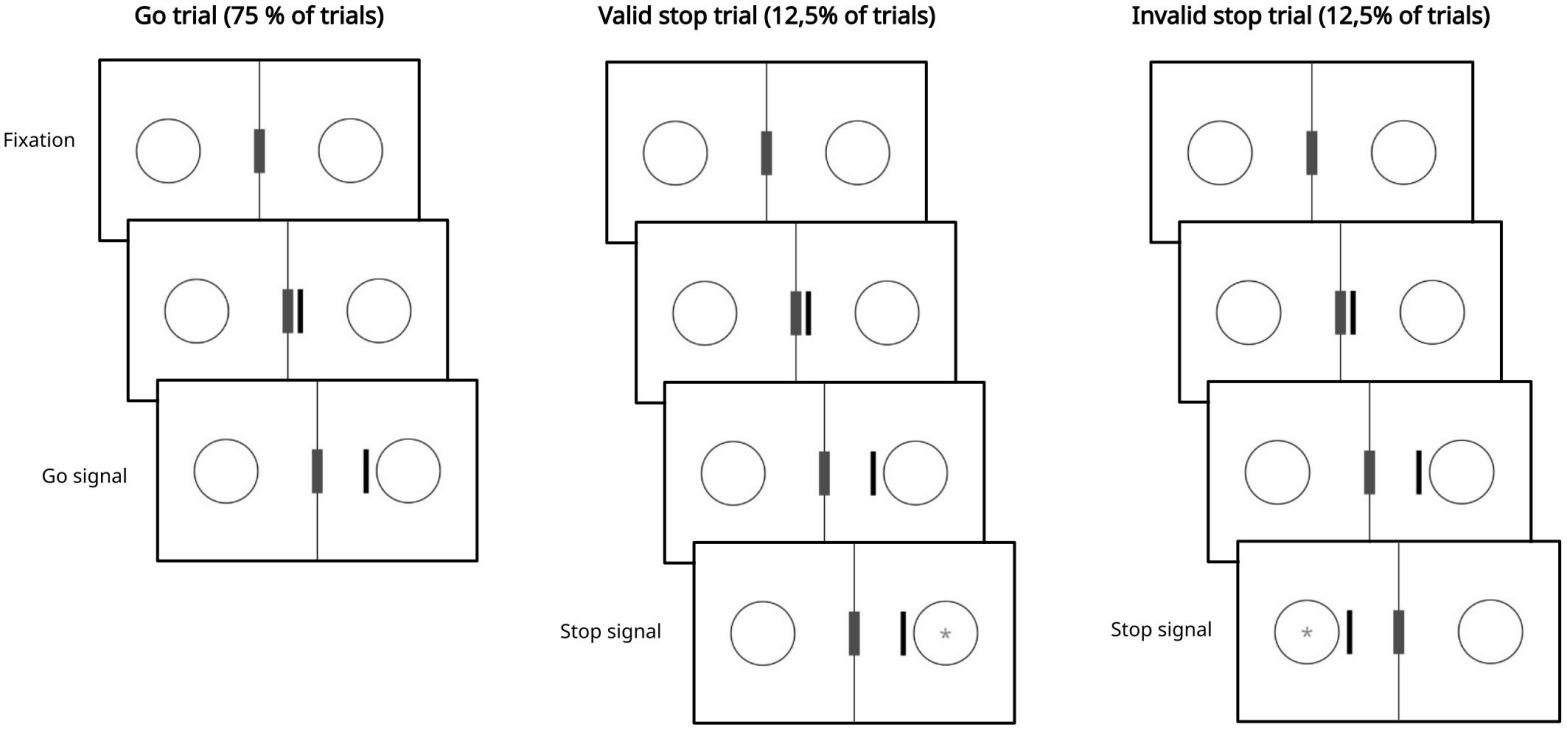
Behavioral task. Trials begin with the presentation of a fixation screen consisting of two circles and a central gray rectangle with a black border on a white background. Subsequently, a black bar appears and starts moving toward one of the two circles in 150 ms steps. When the bar stops near one of the circles, that circle becomes a go signal, and the subject must respond. In stop trials, after a certain interval from the presentation of the go signal, a stop signal (i.e., the gray asterisk within the circle) appears. In this case, participants must attempt to inhibit their response. Stop trials can be classified as valid if the stop signal appears in the circle reached by the black bar, or invalid if it appears in the opposite circle. In the image, the stop signal has been enlarged to make it visible; for its actual size, see the Section 2.3.

In our task (Figure 1), a black vertical bar appeared, moving from the center to the right or left, where circles were positioned. When the bar stopped near one of the circles, it became the go signal. Participants were instructed to respond as quickly as possible by pressing the “K” key. Go trials represented 75% of the total, corresponding to 1,440 trials.

Two types of stop trials were included: valid and invalid. In valid stop trials, the stop signal appeared in the circle reached by the bar, while in invalid stop trials, it appeared in the opposite circle. Based on previous study (Haque et al., 2024), both types of stop signals were presented with four possible SSDs: 100, 200, 300, and 400 ms, with an equal number of trials for each delay. The stop signal was represented by an asterisk that appeared in one of the two circles. A trial was considered correct if the participant refrained from pressing the “K” key during the stop trial; otherwise, pressing the key resulted in an error. Valid stop trials constituted 12.5% of the total, corresponding to 240 trials, as did invalid stop trials, also representing 12.5% of the total, or 240 trials. These two types of trials were presented in equal numbers.

Participants were instructed to fixate on the central gray rectangle while attending the go signal and respond quickly, while inhibiting their response in case of stop signal. Prior to the experimental session, participants underwent a familiarization phase where the task was explained and a total of 64 trials were completed to ensure familiarity with the procedure.

Participants completed a total of 1,920 trials divided into 10 blocks. All participants successfully completed the practice trials before proceeding to the experimental phase. Trials were randomized within each block, with the constraint that no more than two consecutive stop trials could be presented.

### 2.3 Procedure

Data were collected in a soundproof, darkened glass room inside a larger room. Participants were positioned ~50–60 cm from a monitor with a 1,920 × 1,080 resolution and a 60 Hz refresh rate. The experimental setup and data acquisition were implemented using PsychoPy v.2022.2.2 (Peirce et al., 2019) via its experiment builder.

Each trial began with a screen displaying a central gray rectangle (1.3 cm × 5 cm) and two circles with black borders (7.45 cm in diameter) on a white background. The screen remained visible for a random duration between 800 and 1,000 ms. Following this, a black bar (cue, 0.5 cm × 5 cm) appeared and moved toward one of the circles in two steps, each covering 1.5 cm, with intervals of 150 ms between each movement. The total duration of the movement was 300 ms and it consisted of two 150 ms step movements. Finally, this bar stops at 0.85 cm from the target circle (go signal). From this moment, participants had up to 700 ms to respond. This is the temporal sequence for a go trial.

For stop trials, a stop signal (a light gray asterisk, 1.17 cm in diameter) appeared inside one of the circles after the SSD.

At the end of each trial, participants received auditory feedback lasting 500 ms, with a single beep indicating a correct response and two beeps signaling an incorrect response. A white screen without stimuli was presented for one second between trials.

### 2.4 Data analysis

Statistical analyses were performed using MATLAB R2024a software (The MathWorks Inc., Natick, MA). First, to assess whether the independence assumption of the model was met for each participant, a Wilcoxon signed-rank test (Woolson, 2007) was applied. This test enabled a comparison of the reaction time (RT) distributions between go trials and stop trials for each subject. To estimate SSRT, we used the integrative method, which involves replacing go omissions (i.e., go trials with no response) with the maximum RT observed (Verbruggen et al., 2019). The RTs of go trials are first sorted in ascending order, and the number of elements in the RT distribution is multiplied by the response probability for each SSD. The nth RT is then identified, and the corresponding SSD is subtracted from this value to estimate SSRT. SSRT was estimated for all conditions, including both valid and invalid stop conditions and different SSDs, but only for those where the probability of response was between 0.25 and 0.75. The use of these response probabilities in SSRT estimation ensures more precise and reliable calculations (Band et al., 2003; Congdon et al., 2012; Verbruggen et al., 2019).

To evaluate the impact of cueing on response inhibition, we performed analyses on probabilities of response, RTs, and SSRTs. Reaction times were calculated as the time interval between the presentation of the go signal and the participant's response. The probability of response was calculated as the ratio of responses to the stop signal across both valid and invalid stop conditions, with varying SSDs, to the total number of stop trials in each condition. In the calculation of the probability of response, stop trials where the participant responded before the go signal appeared were excluded.

For each experimental condition, the SSRTs were averaged, with separate averages for the valid and invalid stop conditions. ANOVAs were conducted on the SSRTs, RTs, and response probabilities to evaluate the effects of cueing under different conditions. In the SSRT analysis, we focused on “stop validity” as a factor, comparing performance between valid and invalid stop trials. For the RT analysis, the factor was “condition” which included three levels: go trials, valid stop trials, and invalid stop trials. Response probabilities were examined using a two-factor ANOVA, assessing the influence of cueing (valid vs. invalid stop) and SSDs, including four levels: 100, 200, 300, and 400 ms.

To evaluate how the effects of cueing are distributed across our sample, we computed the difference between the probability of response [p(R)] in invalid stops and valid stops for each subject. Specifically, we calculated the difference between p(R) in invalid stops and p(R) in valid stops [i.e., p(R) in invalid stop – p(R) in valid stop] for each SSD. Next, for each SSD, we classified participants based on their performance: those who performed better in invalid stops (negative values in the difference) and those who performed better in valid stops (positive values in the difference). We then performed chi-square tests to examine the distribution of these classifications across participants, conducting separate chi-square analyses for each SSD considered.

## 3 Results

### 3.1 Response times

**Figure 2:**
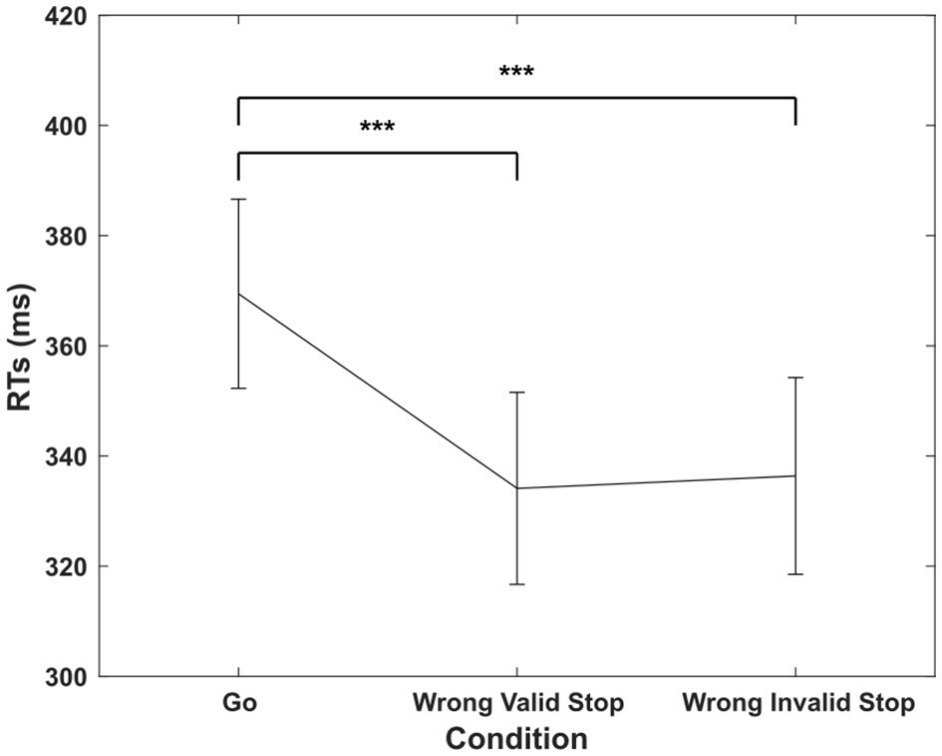
Mean reaction times (in ms) and standard error (± 1 SEM) for the three experimental conditions. Three asterisks indicate a statistically significant difference at *p* < 0.001, as determined by a one-way repeated measures ANOVA conducted on RTs.

We found that RTs in go trials (369.44 ± 17.16 ms) were significantly slower than those in wrong valid stop trials (334.13 ± 17.43 ms) and wrong invalid stop trials {336.38 ± 17.85 ms, ANOVA [F_(2, 30)_ = 79.98, *p* < 0.001, η*p*^2^ = 0.84]; Figure 2}. These results are consistent with the inclusion criteria, i.e., that wrong trials are no longer the go trials for each subject (Independent Assumption). In incorrect stop trials, RTs tend to be shorter, as only the fastest responses are completed before the inhibitory process can be effectively engaged. *Post-hoc* comparisons confirmed significant differences between RTs in go trials and both valid and invalid stop trials (*p* < 0.001 for both, Tukey-Kramer test).

### 3.2 Cueing enhances inhibitory control for invalid stops after 300 ms

**Figure 3:**
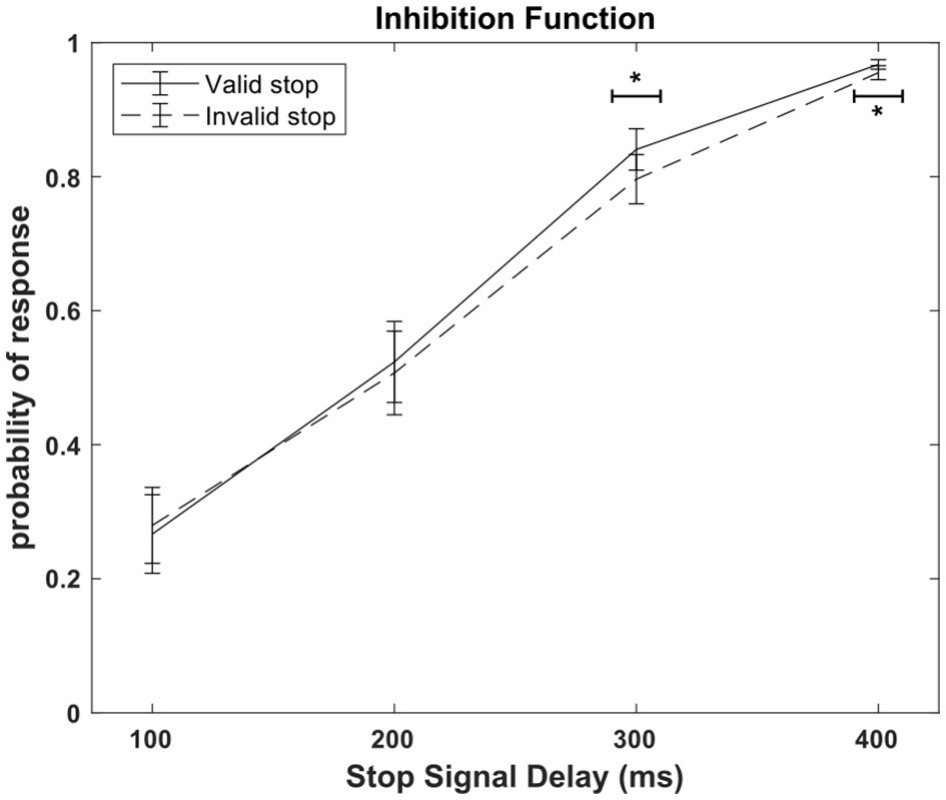
Inhibition function. Probability of response in relation to the stop condition (valid or invalid) and the Stop Signal Delay (100, 200, 300, 400 ms). One asterisk indicate a statistically significant difference at *p* < 0.05, as determined by a two-way repeated measures ANOVA conducted on probability of response.

Figure 3 shows the inhibition function for both valid and invalid conditions, a graphical representation of the probability of response as a function of the Stop Signal Delay. Cueing affected inhibitory control, but this effect was evident to stop signals occurring 300 and 400 ms after the go signal. An ANOVA with the factors SSD and stop validity revealed a significant interaction [F_(3, 45)_ = 3.06, *p* = 0.037, η*p*^2^ = 0.17]. *Post-hoc* comparisons indicated a significant difference in response probability between valid and invalid conditions at the third and fourth SSD. Specifically, in the third SSD, the response probability was lower in the invalid condition (0.79 ± 0.04) compared to the valid condition (0.84 ± 0.03) (*p* = 0.02). Similarly, in the fourth SSD, the response probability was lower in the invalid condition (0.96 ± 0.01) compared to the valid condition (0.97 ± 0.01) (*p* = 0.02), suggesting that inhibitory control is enhanced for invalid stops after 300 ms. The ANOVA also revealed a significant effect of Cueing, F_(1, 15)_ = 4.67, *p* = 0.047, η*p*^2^ = 0.24. The ANOVA also shown a significant main effect of SSD [F_(3, 45)_ = 107.72, *p* < 0.001, η*p*^2^ = 0.88]. As the SSD increased, the response probability also increased, consistent with expected outcomes in the Stop Signal Task paradigm.

### 3.3 Cueing does not affect the SSRT

Following the effect of the interaction between SSD and stop validity on the probability of response, we investigated whether this effect was also evident in the evaluation of the inhibition process latency. We did not find any significant difference between the SSRT for valid stops (288.23 ± 25.14 ms) and invalid stops (301.39 ± 24.69 ms) [F_(1, 14)_ = 1.71, *p* = 0.21] thus showing that overall, the latency of the stopping process is not affected by a valid cueing in a context where valid and invalid signal have the same probability to occur.

### 3.4 The IOR-like effects emerge consistently across individuals

**Figure 4:**
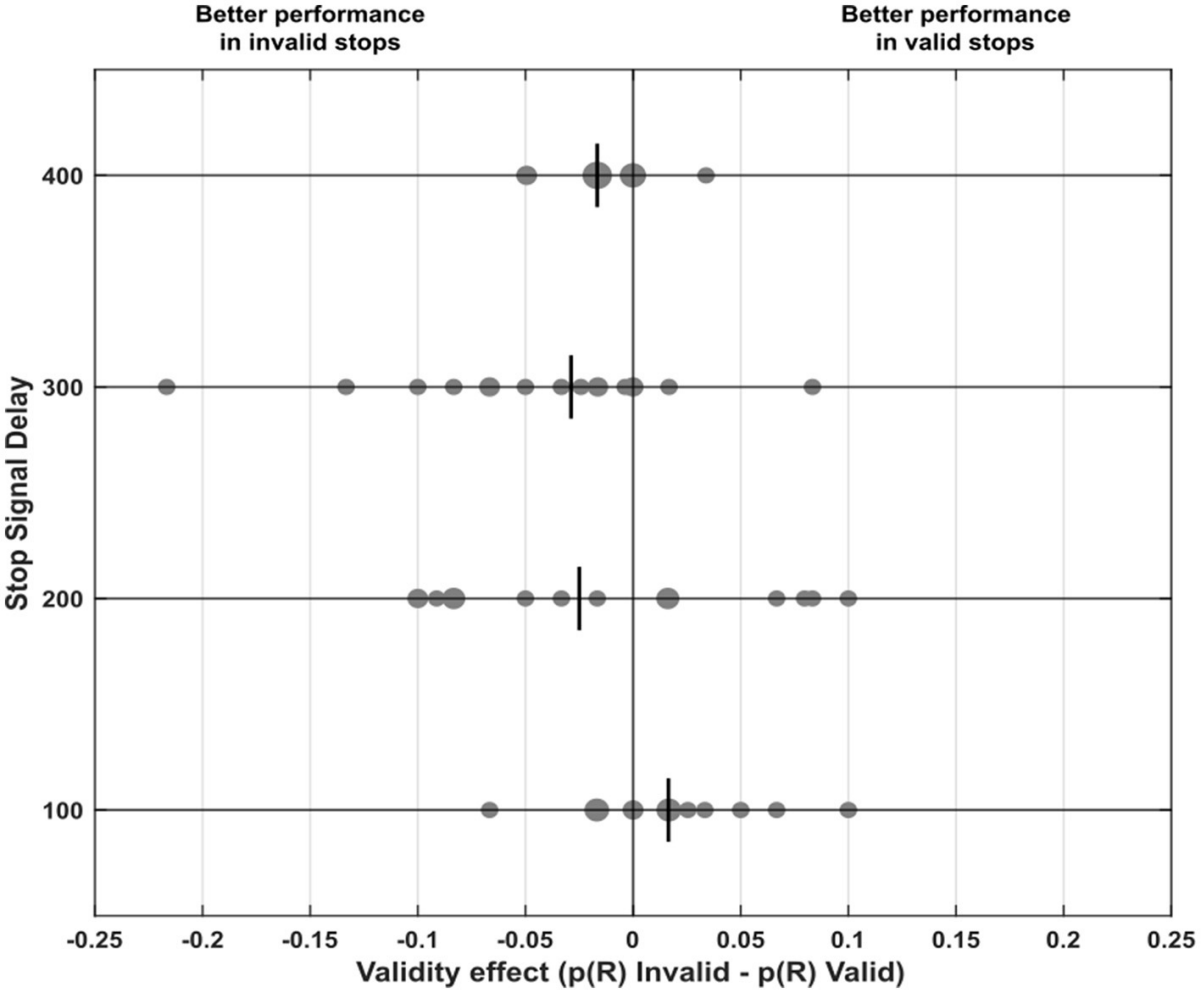
Differences in probability of response (i.e., the probability of response in invalid trials minus that in valid trials of the specific Stop Signal Delay), also referred to as the validity effect, across every Stop Signal Delays (100, 200, 300, 400 ms). Positive values indicate that it was easier to inhibit in valid stop signal trials, whereas negative values indicate that inhibition was easier in invalid stop signal trials. Each small dot represents a single participant, while larger dots highlight clusters of participants with similar values. The vertical lines in each Stop Signal Delay plot represent the median for that specific delay.

Figure 4 illustrates the distribution of the validity effect, calculated as the difference in p(R) between invalid and valid conditions for each SSD across our sample. We found that IOR is consistent. Specifically, for stops occurring after 300 and 400 ms, performance was generally better in the invalid condition: after 300 ms, 12 participants showed better performance in invalid stops, while two performed better in valid stops [*χ*^2^_(1,*N*=14_) = 6.50, *p* = 0.01, *V* = 0.68]; after 400 ms, 11 participants showed better performance in invalid stops, compared to just 1 in the valid condition [*χ*^2^_(1,*N*=12_) = 7.25, *p* =.007, *V* = 0.77].

## 4 Discussion

In the present study, we investigated how non-predictive spatial cueing affects response inhibition. Previous work has shown that factors that modulate attention also alter response inhibition (Verbruggen et al., 2014a; Hilt and Cardellicchio, 2020; Haque et al., 2024); however, how attentional spatial cueing influences motor control is still not well understood.

We employed a Stop-Signal task in which the stop signal was preceded by a non-predictive spatial cue to test the influence of exogenous attention on motor control. Our results revealed no difference between valid and invalid cue conditions in the first 100–200 ms, but a significantly lower probability of responding (i.e., better inhibitory performance) in the invalid compared with the valid stop condition at 300 and 400 ms. This pattern partly mirrors the behavior observed in exogenous Posner tasks (Posner and Cohen, 1984). In these tasks an early advantage for detecting targets at the cued location disappears within 200–300 ms, after which an inhibition-of-return (IOR) effect emerges, facilitating detection of invalid targets (Klein, 2000; Franceschini et al., 2018). In our case we observed that starting from 300 ms, response inhibition was facilitated for invalid cued stop signal, thus suggesting that IOR may influence stop-signal detection, while no difference was observed at 100 and 200 ms.

In our previous study (Haque et al., 2024), employing a similar task, we found that inhibitory performance was better in the valid stop condition than in the invalid stop condition up to 250 ms after the presentation of the go signal. At the longest SSD (450 ms), no difference was observed. An important difference between the present and the previous task is that, in the previous one, the cue correctly predicted the stop signal's location (valid stop trials) in about 70 % of stop trials. The absence of facilitation at early SSDs in this task and its presence in the previous version seems to exclude a role for exogenous attention. Indeed, exogenous deployment of attention is typically observed when the spatial cue is not predictive of the target location, and attention is supposed to be automatically and involuntarily captured and oriented toward the cue location (Macaluso and Doricchi, 2013; Di Bello et al., 2022). At the same time, predictiveness and statistical contingencies are key factors in engaging endogenous attention, or at least top-down biases, that can affect early allocation of resources (Doricchi et al., 2010; Lasaponara et al., 2011; Amengual et al., 2022; Dolci et al., 2024). As such, we could interpret the facilitatory effect in the previous study as driven by top-down processes, although we cannot define whether it was endogenous or related to statistical contingencies (Dolci et al., 2024).

However, in this work, we also observed an IOR at SSDs of 300 and 400 ms, suggesting an involvement of exogenous attention in modulating motor control. Overall, these data suggest that, in our tasks, inhibitory performance at the early SSDs can be affected by different processes.

It has been suggested that when an unexpected event occurs within the context of motor expectations, the motor system may slow down or prevent from responding to reassess the motor plan (Diesburg and Wessel, 2021). In our experiment, both the lack of predictability of the stop signal and its occurrence in only 25% of the total trials likely makes its presentation comparable to an unexpected event, possibly causing a broad motor interruption with no differences between valid and invalid cues (Wessel and Aron, 2017). This generalized inhibitory response is thought to be short-lived (~150–200 ms; Wessel and Aron, 2017; Giarrocco et al., 2021; Hannah et al., 2022; Pani et al., 2022b). As such, the influence of spatial attention should become evident at longer SSDs, around 300 ms or more. Consistent with this idea, we observed as IOR-like effect at longer SSDs (300–400 ms), showing improve stopping performance in invalid trials. Diverse pieces of evidence indicate that exogenous attention, unlike endogenous attention, has a strong connection with the motor system (Hunt et al., 2019; Smith and Schenk, 2012; Xia et al., 2024; Di Bello et al., 2024), including areas involved in motor inhibition. Indeed, the right inferior frontal gyrus (rIFG) is shared by the ventral attention network and the fronto-basal ganglia network, which governs inhibitory control. This region plays a crucial role in stopping ongoing actions in response to a stop signal or an unexpected event (Aron et al., 2014). Thus, combining the current observation with the previous investigations, we can hypothesize that the specific attention (or global stopping process) at play depends on the task structure. Looking at the facilitatory effect observed in Haque et al. (2024), we can suggest that cue predictiveness may play a key role in shaping inhibitory strategies—not only by enhancing perceptual processing of the stop signal but also by preventing global stopping.

This is consistent with previous studies indicating that exogenous and endogenous attention, though functionally distinct, can interact dynamically depending on task demands (Egeth and Yantis, 1997; Carrasco, 2011; Di Bello et al., 2024). It is also worth considering that the task's low visual-processing demands (i.e., visual detection) may render endogenous attentional deployment non-mandatory to solve the task. As highlighted in Treisman and Gelade's (1980) seminal work, simple stimulus detection may occur without active attentional engagement, unlike more complex visual processes such as target discrimination or identification. Based on this, the detection of the go signal may primarily rely on exogenous shifts at the moment of the bar's stop, thus avoiding unnecessary attentional effort (Carrasco, 2011). The evidence that exogenous attention can be elicited even by minimally salient events, such as peripheral cue offsets (Riggio et al., 1998), is consistent with this interpretation. Further investigations that isolate exogenous attention will be necessary to clarify this aspect. A reduced effect of attention on motor control could also result from reduced deployment of exogenous attention in the proposed task. Peripheral cues are generally expected to attract attention exogenously due to their salience, even when they are not spatially predictive of the target's position. In Posner-like protocols, these cues typically coincide with the locations where targets appear, offering participants a single spatial reference for processing both the cue and the target. In our experiment, however, this perfect coincidence was absent, potentially reducing the attentional-capture ability of these peripheral stimuli. Nevertheless, previous studies have demonstrated that stimuli not aligned with the target placeholders can still effectively orient attention (Chica et al., 2014). However, other factors can be at play and will need to be evaluated. For instance, in our previous study, the stop trials were ~40% of the total trials, whereas in the current study, this percentage was reduced to 25%, thus possibly affecting more general behavioral strategies (Andujar et al., 2024; Bissett and Logan, 2011). Furthermore, we expanded the set of SSDs, increasing the number from three to four. The impact of this change on inhibitory performance remains unclear.

Our investigations contribute to the increasing number of studies have explored how attentional factors influence inhibitory capacity (Verbruggen et al., 2014b). For instance, in a context where detecting the stop signal is more challenging due to the presence of distractors than in a context without distractors, the inhibitory process becomes more difficult, resulting in an increased SSRT (Verbruggen et al., 2014a). The salience of the stop signal can impact inhibitory control (van der Schoot et al., 2005; Morein-Zamir and Kingstone, 2006; Camalier et al., 2007; van Gaal et al., 2009; Montanari et al., 2017), while the visual gap (fixation disappearance) can prolong the SSRT, with effects varying depending on the motor system involved (Mirabella et al., 2009; Stevenson et al., 2009).

Studying the relationship between attentional processes and inhibitory control can have implications for daily life. For instance, in situations where dangers must be signaled, the use of exogenously capturing visual cues may not be the best solution—especially if a signal indicating the need to stop a motor action (such as halting the operation of industrial machinery in response to a safety alert) is presented at the same location. In such cases, the attentional capture could interfere with the proper inhibition of the motor response, potentially compromising safety.

Although further research is needed, our results contribute to the understanding of the contribution of exogenous and endogenous attention to motor control. Building on this topic will be also crucial in clinical contexts, where tasks targeting cognitive stages help to test hypotheses about specific disorder showing both attentional and motor control deficits (Lampe et al., 2007; Alderson et al., 2008; Senderecka et al., 2012; Salum et al., 2014; Matzke et al., 2017).

## Data Availability

The raw data supporting the conclusions of this article will be made available by the authors, without undue reservation.
